# Human Auditory and Adjacent Nonauditory Cerebral Cortices Are Hypermetabolic in Tinnitus as Measured by Functional Near-Infrared Spectroscopy (fNIRS)

**DOI:** 10.1155/2016/7453149

**Published:** 2016-03-02

**Authors:** Mohamad Issa, Silvia Bisconti, Ioulia Kovelman, Paul Kileny, Gregory J. Basura

**Affiliations:** ^1^Department of Otolaryngology/Head and Neck Surgery, Kresge Hearing Research Institute, The University of Michigan, 1100 W Medical Center Drive, Ann Arbor, MI 48109, USA; ^2^Center for Human Growth and Development, The University of Michigan, 1100 W Medical Center Drive, Ann Arbor, MI 48109, USA

## Abstract

Tinnitus is the phantom perception of sound in the absence of an acoustic stimulus. To date, the purported neural correlates of tinnitus from animal models have not been adequately characterized with translational technology in the human brain. The aim of the present study was to measure changes in oxy-hemoglobin concentration from regions of interest (ROI; auditory cortex) and non-ROI (adjacent nonauditory cortices) during auditory stimulation and silence in participants with subjective tinnitus appreciated equally in both ears and in nontinnitus controls using functional near-infrared spectroscopy (fNIRS). Control and tinnitus participants with normal/near-normal hearing were tested during a passive auditory task. Hemodynamic activity was monitored over ROI and non-ROI under episodic periods of auditory stimulation with 750 or 8000 Hz tones, broadband noise, and silence. During periods of silence, tinnitus participants maintained increased hemodynamic responses in ROI, while a significant deactivation was seen in controls. Interestingly, non-ROI activity was also increased in the tinnitus group as compared to controls during silence. The present results demonstrate that both auditory and select nonauditory cortices have elevated hemodynamic activity in participants with tinnitus in the absence of an external auditory stimulus, a finding that may reflect basic science neural correlates of tinnitus that ultimately contribute to phantom sound perception.

## 1. Introduction

Tinnitus is the perception of sound in the absence of an extraneous sound source [1]. This disorder is highly prevalent around the world, with an estimated 10–15% of US adults suffering from tinnitus [2]. One subgroup that is particularly affected by this condition is military personnel. Tinnitus is the number-one service related disability, with over 744,000 total veterans receiving disability compensation at a cost to American taxpayers over $2.26 billion in 2014 [3].

The underlying etiology of tinnitus is not well-defined, yet it is typically associated with many forms of peripheral ear pathology, hearing loss, retrocochlear lesions, head and neck injury, dental and temporal-mandibular joint dysfunction, and drug toxicity [4]. Changes at the auditory periphery often lead to aberrant neural activity within central auditory pathways that may be localized within and influenced by multiple brain stations. As a result, a central etiology of tinnitus has been supported by persistence of phantom sound perception following auditory nerve transection [2].

Animal studies have been instrumental in elucidating potential central neuronal mechanisms of tinnitus. Following noise damage, increased spontaneous and tone-evoked neural firing rates and enhanced neural synchrony have been found at multiple brain stations including primary auditory cortex [5]. These putative neural correlates of tinnitus have largely been unexplored or translated within human central auditory pathways. One reason for this is due, in part, to the limitation of technology in capturing human central auditory circuits under “real time” conditions of aberrancy. Optical imaging modalities used to study tinnitus include functional magnetic resonance imaging (fMRI), positron emission tomography (PET) with and without computer tomography (PET-CT), electroencephalography (EEG), and magnetoencephalography (MEG). Meaningful data captured from these seemingly noninvasive imaging modalities have been limited by the potential confounding effect of extraneous noise (MRI), use of high production-cost radioisotopes for PET studies, and limited spatial resolution for EEG and MEG studies [6, 7]. Although there are limitations to each imaging modality, reported results with current technology have identified changes in tinnitus brains that may reflect basic science neural correlates as described in animal models [8, 9]. Additional optical image technologies are needed to provide an alternative means to potentially further translate these putative correlates within the central human auditory pathway.

Functional near-infrared spectroscopy (fNIRS) is an imaging modality that has emerged as a noninvasive metric of cortical hemodynamic activity in many human auditory and nonauditory studies [10, 11]. This technology takes advantage of the changing optical properties of brain tissue using near-infrared light to extrapolate and quantify hemodynamic responses through neurovascular coupling, whereby activated brain regions trigger increased blood flow that can be captured and monitored for changes in oxygenated and deoxygenated hemoglobin [10]. When a specific brain region is activated, fNIRS measure changes in localized hemoglobin level as an index of neural activity within the specific region. By relying on the intrinsic optical properties of blood, fNIRS provides a more direct metabolic marker relative to the widely used BOLD effect in fMRI, which derives contrast only from the paramagnetic properties of deoxyhemoglobin [12].

fNIRS has reliably confirmed increased and decreased hemodynamic activity in auditory and surrounding nonauditory regions under conditions of acoustic stimulation [13–16] and during rest/silence [17], respectively. Plichta et al. [13] recorded brain activation with fNIRS while participants listened to pleasant, unpleasant, and neutral sounds and observed that unpleasant sounds increased auditory cortex activation when compared to neutral sounds. Alternatively, the utility of fNIRS to calculate temporal synchronization of spontaneous neuronal activity within various cortical regions, referred to as resting-state functional connectivity, has also been reported [17].

To the best of our knowledge, only one prior study has used fNIRS technology to investigate tinnitus in humans [18]. That proof of concept study demonstrated the utility of fNIRS to detect levels of oxygenation in auditory cortex in participants with tinnitus at rest and in response to transcranial magnetic stimulation (TMS). Interestingly, participants with tinnitus demonstrated a decrease in hemodynamic activity following TMS suggesting that increased auditory cortical activity as measured by fNIRS may be related to tinnitus perception. Schecklmann et al. [18] demonstrated higher oxygenation at baseline over the right auditory cortex in participants with tinnitus than in controls. The authors concluded that this increased activity is at least partially representative of the tinnitus percept and validated the use of fNIRS as an investigative tool to study the pathophysiology and treatment response.

The hypothesis of the present study is that participants with tinnitus and essentially normal hearing will show increased hemodynamic responses under conditions of silence indicative of physiologic neural correlates including increased spontaneous neural firing rates in animal models. Our hypothesis using a randomized auditory stimulus and silence block paradigm will extend the current understanding of hemodynamic changes that occur in human tinnitus brains using fNIRS technology as published [18]. To test this hypothesis, we created two analytical approaches, one that involves a standard measurement of hemodynamic activation during auditory stimulation after subtracting the initial periods of stimulation (thereby avoiding baseline activations in the analysis) and secondly by measuring hemodynamic responses during intervening periods of silence. Here we demonstrate that during periods of silence hemodynamic responses are maintained in auditory and adjacent nonauditory cortices in tinnitus participants, a response not seen in controls. Interestingly, the elevated hemodynamic states during silence are subsequently decreased with broadband noise in tinnitus indicative of forward masking and residual inhibition. While these data derived from fNIRS waveforms may not be directly representative of putative neural physiologic correlates of tinnitus from animal models, measurable changes using this innovative technology may provide a translational index of activation seen in auditory and surrounding associative auditory cortices.

## 2. Material and Methods

### 2.1. Participants

The University of Michigan Institutional Review Board approved the study and participants were reimbursed for participation. Informed consent was obtained from each participant after an extensive explanation of the protocol using noninvasive fNIRS technology. Ten normal/near-normal hearing adults with subjective tinnitus (average age: 48.7 ± 16 years) appreciated equally in both ears and seven normal/near-normal hearing nontinnitus controls (average age: 25.7 ± 7.8 years) participated in the study. The selection of normal and near normal hearing tinnitus participants was based on the desire to rule hearing loss out as a factor influencing the fNIRS results. All of the participants suffered from constant, nonpulsatile tinnitus percepts that were appreciated in the “head” or in both ears equally. None of the participants endorsed concurrent hyperacusis (hypersensitivity to sound). Exclusion criteria included prior otologic surgery, unilateral tinnitus, and any degree of conductive hearing loss, sensorineural hearing loss greater than 30 dB at any frequency or other identified etiologies of tinnitus (i.e., glomus skull base tumors, retrocochlear lesions, and high dose aspirin). In order to maximize the ability to compare the two groups, participants were enrolled only if they demonstrated bilateral normal/near-normal hearing thresholds confirmed via an audiogram. Both control and tinnitus participants were held to the same normal/near-normal hearing criteria defined as hearing thresholds less than 30 dB for pure tone averages (PTA) across all frequencies, including high-frequency regions such as 8000 Hz. Moreover the speech reception thresholds (SRTs) and word discrimination scores (WDS) were also found to be within the normal range between both groups. The SRT is defined as the lowest threshold (in dB) at which 50% of spoken spondees (balanced two syllable words) are correctly identified for each ear. The WDS is based on the percentage of single-syllable words correctly understood when stimuli are presented at a normal conversational speech level (40–60 dB). Prior to testing, individuals with tinnitus completed a subjective tinnitus assessment scale (Tinnitus Handicap Inventory; THI; [19]) to quantify and qualify the severity of tinnitus by evaluating both the emotional burden and degree of daily disruption caused by the condition.

### 2.2. fNIRS Imaging

**Figure 1:**
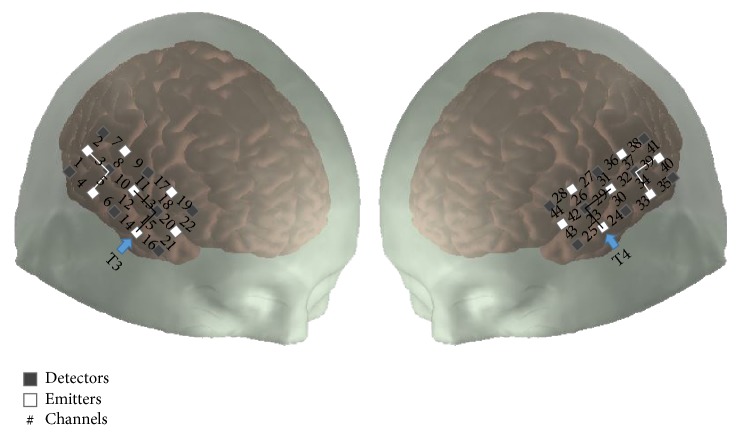
Configuration of channels (numbers) with identified detectors (dark gray squares) and emitters (white squares) over the right and left cortical hemispheres. There are 8 detectors and 7 sources resulting in 22 channels per hemisphere. The unit of concentration is micromolar (*μ*M). Interconnected blocks with the solid black line represent the region of interest (ROI; primary auditory regions; channels 13, 15, 23, and 29). Interconnected blocks with white line represent the nonregion of interest (non-ROI; (channels 3, 5, 34, and 39)). T3 and T4 are the reference points of the International 10–20 System [21].

We used a continuous wave fNIRS system (CW6, Techen, Inc., USA) with two wavelengths (690 nm and 830 nm).A customized configuration of 30 optodes (15 per hemisphere; source-detector pairs; Figure 1) inserted into a silicone band was wrapped circumferentially around the head and secured with Velcro straps. There are 8 detectors and 7 sources resulting in 22 channels per hemisphere. Emitters and detectors were arranged into 5 × 3 arrays over the frontal, temporal, and parietal cortices of the right and left hemispheres. The distance between each light emitter and detector was set at 3 cm. To identify if the probe/optode array had moved during testing, the positions of T3 and T4 were confirmed before and after experiment. Additionally, postexperiment photographs were taken and reviewed for each participant to ensure consistent probe location. The data were collected at a sampling rate of 20 Hz. Since fNIRS measures light intensity several conversation steps were made between the captured intensity values to derive the final data measured in units of micromolar (*μ*M) concentration [20].

### 2.3. fNIRS Anatomical Localization/ROI Selection

The region of interest (ROI) included primary auditory cortex (temporal lobe including superior temporal plane) and surrounding auditory belt regions (temporal and parietal cortices). Acknowledging the spatial resolution limits of fNIRS technology, both anatomical (10–20 EEG system) and functional (normal brain response to auditory stimulation) strategies were utilized to identify ROI. First, the International 10–20 System for EEG electrode placement [22] with bilateral T3/T4 coordinates for temporal optode placements (Figure 1; [13, 23]) was utilized. Second, anatomical localization of ROI was achieved by isolating only those channels that in control participants showed an increased average group hemodynamic activity in response to auditory stimuli and subsequent declines in the presence of silence as previously reported [13–17, 23]. The ROI (and non-ROI; see below) were separately derived from 2 respective channels from each hemisphere that were then averaged to obtain the final concentration for all test conditions. Because there were no statistically derived differences between the hemispheres in all participants, the data was averaged and the ROI corresponded to channels 13 and 15 (right hemisphere) and 23 and 29 (left hemisphere; Figure 1).

To discern whether brain regions not directly associated with auditory processing were activated during periods of auditory stimulation and silence, a nonregion of interest (non-ROI) was selected using published criteria [13]. The non-ROI covered the area immediately outside/adjacent to ROI minus an additional channel (*n* − 1) over the occipital region, which has known functional relationships with the auditory sensory systems [24–27]. The channels corresponding to the non-ROI were 3 and 5 (right hemisphere) and 34 and 39 (left hemisphere; Figure 1). Based on that selection criteria channels were identified auditory (ROI) and nonauditory (non-ROI) cortical regions that equated to Brodmann areas 19 and 37.

### 2.4. Stimuli Protocol

**Figure 2:**
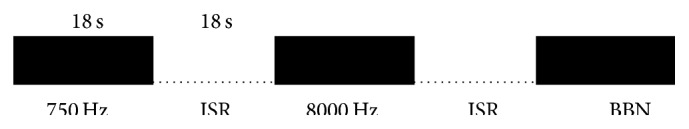
Schematic of block auditory testing paradigm. Participants passively listened to randomly selected pure tones (750 Hz or 8000 Hz) or broadband noise (BBN) for 18 sec each, immediately followed or preceded by an interstimulus rest period (ISR) consisting of silence/absence of auditory stimulation for 18 sec for a total experiment run time of 20 minutes. Each randomized paradigm was repeated 9 times to prevent potential confounding effects and to optimize average responses under each recording condition.

Participants were engaged in a passive listening block paradigm that consisted of periods of silence (interstimulus rest; ISR), broadband noise (BBN), or tonal (750 or 8000 Hz) stimuli using Audacity (GNU General Public License) and normalized with Praat 4.2 [28]. Auditory stimuli were presented via E-prime (Psychology Software Tools, Inc., Pittsburgh, PA, USA) and played at a standard fixed volume through two loudspeakers placed at a distance approximately 2 feet from the subject in a sound field orientation that was held constant for all participants at 70 dB sound pressure level (SPL; Creative Inspire T12). This configuration achieved a consistent SPL given participant selection criteria that included a SRT range of 10–20 dB and comparable pure tone averages (PTA). Thus the SPL level was well within the participants auditory detection range. Our tinnitus participants were matched to controls, including hearing levels. Participants were positioned at arm's length from a desk mounted computer monitor with a projected “plus sign” image to maintain stable head position (without formal head fixation/rest platform) through visual fixation to prevent motion artifact during the recording session. Participants were instructed to stay awake and to simply sit and listen with their gaze fixed on the screen. Participants were initially presented with a sample of each of the testing condition stimuli as a demonstration prior to the start of the formal research paradigm. The auditory testing paradigm consisted of 9 rounds of randomly chosen 18-second blocks of 750 Hz, 8000 Hz, or BBN separated by intervening 18-second blocks of silence (interstimulus rest; ISR) between each auditory stimulus (Figure 2). Each experimental run per participant consisted of 54 blocks: 27 silent (ISR) and 27 stimulation blocks equally distributed amongst the three experimental stimuli (750 Hz, 8000 Hz, or BBN). Auditory stimuli were selected to evaluate hemodynamic responses during partial (750 Hz and 8000 Hz) and complete (BBN) auditory cortical tonotopic activation as compared to ISR in the two groups [29]. The order of auditory stimuli presentation was randomized across the 20-minute experiment.

### 2.5. Data Analysis

All data were preprocessed using Homer2 software [30] based on MATLAB (Mathworks, MA, USA). Raw optical intensity data series were initially converted into changes in optical density. The E-Prune channel function was used to exclude channels with very low optical intensity from analyses [31]. In addition, to remove motion artifact from the data, the wavelet-based motion artifact was used [32]. The parameter *α* was set to 0.1 [31–33]. The data was low-pass filtered at 0.5 Hz in order to eliminate physiological fluctuations and high-pass filtered at 0.01 Hz to remove instrumental noise. Optical density data was then converted into concentration changes using a partial path length factor of 6.0. Since the hemodynamic response following auditory stimulation takes approximately 4–6 seconds to reach maximum level [30], the preprocessed signal was averaged across all blocks over a time range of 4–18 seconds for all conditions. Normalization of the hemodynamic activity was achieved by subtracting the mean value of the first 3 seconds from the block hemodynamic activity average. This was done in order to prevent capture of any dynamic changes occurring in the immediate preceding block where at the end of the recording block the hemodynamic activity was still elevated. This procedure was conducted in each condition and group for oxy-hemoglobin (HbO) and deoxy-hemoglobin (HbR), separately [30, 34]. Channels covering the ROI and non-ROI were subsequently used for the statistical analyses. Standard deviations were calculated using the equation: $\sigma =\surd (\Sigma {\left(a-\overset{-}{a}\right)}^{2})/N$ (with *σ* = standard deviation; *a* = each value in the population; $\overset{-}{a}$ = the mean of the values; *N* = the number of values). Standard errors were calculated using the equation: *σ*_*x*_ = *S*/√*N* (*S* = sample standard deviation; *N* = the number of values).

### 2.6. Statistical Analyses

Statistical analyses were focused on the oxygenated hemoglobin (HbO) signal because HbO constitutes a greater portion of signal from the cortex (76%) compared to deoxy-hemoglobin (HbR; 19%; [35]). Moreover, HbO is sampled over a larger region of brain tissue, and the signal-to-noise contrast for HbO is better than HbR [36]. The correlations between the canonical model of the hemodynamic response function and models of HbO (versus HbR) are consistently higher [30]. Thus, we used HbO as a more robust index of underlying neural activity.

The authors conducted testing of normality (Kolmogorov-Smirnov test) for the variables of interest including metabolic activity per group in the various experimental conditions. The results indicated normal data; those results along with normal appearing histogram and boxplots facilitate the use of parametric tests. Pearson correlation analyses were conducted between age, hearing threshold, and Tinnitus Handicap Inventory (THI) scores with hemodynamic responses during the various experimental paradigms (ISR, BBN, 750 Hz, and 8000 Hz) in both ROI and non-ROI. To assess for interhemispheric differences for each test condition and to rule out the possibility of optode asymmetry paired *t*-test comparisons between right and left ROIs and non-ROIs within specific experimental conditions were conducted. These analyses showed no interhemispheric differences and enabled hemodynamic responses to be pooled for each participant for final evaluation.

In order to examine whether HbO differences existed between conditions, ROIs, and groups, a 2 × 4 × 2 repeated measures analysis of variance (ANOVA) with Bonferroni correction was conducted (IBM SPSS Statistic 21; SPSS, Inc., Chicago, IL, USA; significance at *p* < 0.05). The ANOVA allows for post hoc analyses to investigate the neurophysiological response to sound within group, ROI, and condition. Thus, we performed paired *t*-test analyses within each of the experimental groups between the HbO mean value following each auditory stimulation and ISR in ROI and non-ROI. This allowed us to verify if tinnitus and control participants showed activation to the auditory stimuli, separately. Paired *t*-tests were then conducted between the HbO mean values of ROI and non-ROI for each type of auditory stimulation (750 Hz, 8000 Hz, and BBN). This allowed us to verify if the response was limited to the auditory regions. Following this, we performed independent *t*-test analyses to assess hemodynamic activity differences during the various experimental conditions, including ISR.

## 3. Results

### 3.1. Behavioral Data Analyses

Only those participants with normal/near-normal hearing were included in the study. Within controls the average SRT was 15 dB with an average WDS of 99%, while the tinnitus group had an average SRT of 19 dB and 100% WDS. Independent *t*-tests were conducted to assess differences in audiogram results between tinnitus and control participants. The analysis indicated no differences in hearing thresholds between the two groups (*p* = 0.10). To characterize the severity of tinnitus symptoms across the group, participants completed the THI. The results demonstrate a broad range of tinnitus severity. Two participants were grade 1 (slight severity easily masked), two at grade 2 (mild severity easily masked; occasionally interferes with sleep), and one at grade 3 (moderate severity difficult to mask) with the final four participants scoring grade 4 (severe severity always heard; altered sleep). None of the participants in the study scored a grade 5 (catastrophic severity always heard; interfere with sleep and daily activities). Pearson correlation analysis was conducted between age, hearing threshold, THI score, and audiogram findings with hemodynamic activity during the various experimental paradigms (ISR, BBN, 750 Hz, and 8000 Hz) in both ROI and non-ROI. The only significant effect was a negative correlation between THI score and hemodynamic activity in non-ROI during 750 Hz tone stimulation (Pearson correlation of −0.7; *p* = 0.02). No other correlation approached statistical significance.

### 3.2. Hemispheric Differences

None of the participants were excluded from the study due to inadequate fNIRS signal or changes in headband position as determined by pre- and postexperimental photographs. Paired *t*-tests on the right and left hemispheres within specific experimental conditions pooled across the length of the experiment did not reveal any significant differences. In the tinnitus group, a trend of increased hemodynamic activity in the left hemisphere was seen in response to BBN in both ROI (*t* = −1.80; *p* = 0.79) and non-ROI (*t* = −1.95; *p* = 0.67) but was not significant. Paired *t*-tests analysis between pooled right and left hemispheric hemoglobin concentration across the experimental paradigm revealed a trend of left greater than right brain hemodynamic response in both groups in both ROI and non-ROI that was not significantly different (ROI control: right mean 0.08 ± 0.49; left mean 0.31 ± 0.39; non-ROI control: right mean −0.96 ± 0.70; left mean −0.70 ± 0.48; ROI tinnitus: right mean −0.28 ± 0.34; left mean −0.09 ± 0.29; non-ROI tinnitus: right mean −0.72 ± 0.41; left mean −0.58 ± 0.27; *p* > 0.05). The handedness of the participants was not determined and this variability may account for this observed trend. No other interhemispheric differences or trends were identified in either group. Given the lack of interhemispheric asymmetry, all remaining analysis pooled total right and left hemispheric data for ROI and non-ROI analysis.

### 3.3. Increased Activity in Tinnitus at Rest

**Figure 3:**
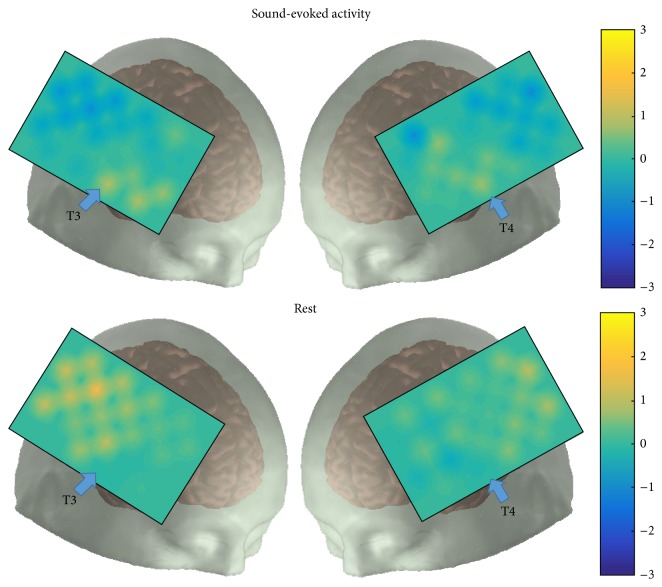
Sound-evoked activity (upper panel) versus responses during silence/rest (lower panel) recorded between right (left hand column) and left (right hand column) hemispheres in control brains. Localization of light emitters and detectors (as mapped in Figure 1) reveals greater activity (yellow) over auditory cortex during acoustic stimulation corresponding to ROI versus less activity over non-ROI (blue). During periods of rest (ISR) note the reversal of brain activity between ROI and non-ROI.

**Figure 4:**
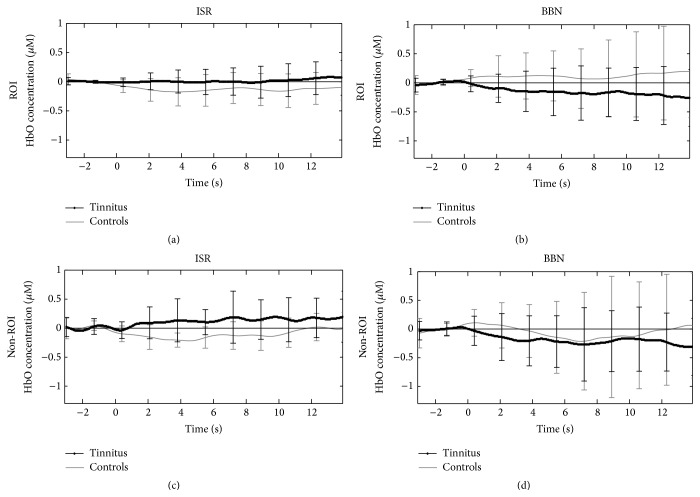
Mean oxy-hemoglobin (HbO) concentration traces versus time (seconds) from ROI and non-ROI under conditions of silence (interstimulus rest; ISR) and stimulation (broadband noise; BBN) between tinnitus and control groups. The unit of concentration is micromolar (*μ*M). Standard deviations at each time point of the traces were calculated using the equation: $\sigma =\surd (\Sigma {\left(a-\overset{-}{a}\right)}^{2})/N$ with *σ* = standard deviation; *a* = each value in the population; $\overset{-}{a}$ = the mean of the values; *N* = the number of values. The figure represents brain activity during the 4–18-second time period in the respective testing paradigm. In the figure, 0 corresponds to 4 seconds of the 18 sec period. Left hand column demonstrates maintained or increased HbO concentrations in tinnitus participants (dark lines) relative to control (gray lines) in both ROI (a) and non-ROI (c) under conditions of silence (ISR). Note the deactivation in controls in both regions during ISR. Right hand column demonstrates the effects of BBN on HbO concentration in ROI (b) and non-ROI (d) in both groups. Note the robust suppression of HbO in tinnitus in response to BBN that continues throughout the recording block ((b) compared to (a)) with the as expected increases in control HbO indicative of auditory stimulation in the intact circuit. BBN also suppresses HbO in non-ROI ((d) compared to (c)) that is also maintained across the recording block suggesting residual inhibition in nonauditory cortical regions in tinnitus subjects. All traces are marked with standard errors calculated for the 4 channels (2 on the right and 2 on the left for both ROI and non-ROI).

During ISR, controls demonstrated an as expected significant deactivation of HbO within ROI during the entire interval, suggesting that hemodynamic activity decreased with the cessation of auditory stimulation (Figures 3 and 4(a)). Conversely, tinnitus participants maintained or slightly increased HbO concentration throughout the recording block during ISR (Figure 4(a)). *t*-test comparisons revealed that controls had a larger HbO decrease than tinnitus (*t* = −2.9; *p* < 0.001), suggesting that tinnitus participants have greater ROI metabolic activity during periods of silence/absence of acoustic stimulation.

Interestingly, tinnitus participants also demonstrated maintenance of hemodynamic responses (increased HbO) within non-ROI during ISR (Figure 4(c)) indicating that central tinnitus origins may also exhibit physiological changes over other regions of brain not primarily dedicated to auditory function. *t*-test comparisons demonstrated that controls had more significant HbO decreases than tinnitus over the non-ROI (*t* = −2.5; *p* = 0.01), indicating, as in the ROI, that tinnitus participants have greater non-ROI metabolic activity at rest.

### 3.4. Neurophysiological Responses to Sound

The results of the ANOVA demonstrated a main effect for brain region (*F*_(1,68)_ = 10.80; *p* < 0.001) and revealed a significant interaction between the various experimental conditions (ISR, BBN, 750 Hz, and 8000 Hz) and region of analysis (ROI versus non-ROI; *F*_(3,66)_ = 4.78; *p* = 0.03) indicating that, as expected, the two regions had different hemodynamic responses under various experimental conditions (Table 1). A significant interaction was found between the various experimental conditions and the two groups (*F*_(3,66)_ = 4.80; *p* = 0.01), indicating that tinnitus and control participants responded to the experimental conditions differently. Post hoc analyses revealed that control participants showed significant HbO increases during 750 Hz, 8000 Hz, and BBN when compared to the periods of silence in the ROI, corresponding to the area of the auditory cortex (750 Hz, *t* = 2.60, *p* = 0.01; 8000 Hz, *t* = 2.10, *p* = 0.04; BBN, *t* = 2.30, *p* = 0.03; Table 1). As expected, in the non-ROI, control participants did not demonstrate any changes in hemodynamic activity during tone (750 Hz, 8000 Hz) or noise (BBN), indicating that this region was not involved in auditory processing (750 Hz, *t* = 0.80, *p* = 0.40; 8000 Hz, *t* = 0.60, *p* = 0.50; BBN *t* = −0.10, *p* = 0.90). These results indicate that our data are plausible with neurophysiological response to sounds.

Following stimulation with BBN, HbO concentrations that were maintained in the tinnitus group under conditions of silence showed a significant deactivation in ROI relative to ISR (*t* = −2.2, *p* = 0.04; Figure 4(b)). The suppressed activity in response to BBN in ROI was maintained and actually continued to decline over time during the experimental condition suggesting long-term effects likely consistent with forward masking and residual inhibition of response to broad auditory stimulation (Figure 4(b)). Like the effects on ROI, BBN also interestingly lead to a significant deactivation in measured HbO over the non-ROI as compared to ISR (*t* = −3.30, *p* < 0.001; Figure 4(d)).

**Figure 5:**
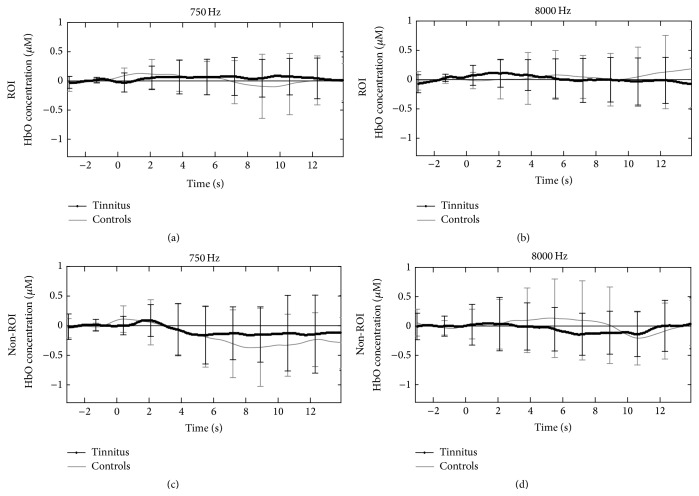
Mean oxy-hemoglobin (HbO) concentration traces versus time (seconds) from ROI and non-ROI under conditions of tonal auditory stimulation (750 Hz and 8000 Hz) between tinnitus (dark lines) and control (gray lines). The unit of concentration is micromolar (*μ*M). Standard deviations at each time point of the traces were calculated using the equation: $\sigma =\surd (\Sigma {\left(a-\overset{-}{a}\right)}^{2})/N$ with *σ* = standard deviation; *a* = each value in the population; $\overset{-}{a}$ = the mean of the values; *N* = the number of values. The panels represent brain activity during the 4–18-second time period during the respective experimental paradigm. In the figure, 0 corresponds to 4 seconds of the 18-second period. Left hand column demonstrates responses to 750 Hz in both ROI (a) and non-ROI (c). Despite variable responses no significant differences are seen between tinnitus and control. Right-hand column demonstrates responses to 8000 Hz in ROI (a) and non-ROI (b) in both groups. Again, despite variability in traces, no significant differences are seen between tinnitus and control. All traces are marked with standard errors calculated for the 4 channels (2 on the right and 2 on the left for both ROI and non-ROI).

Although a trend of HbO increase was found for 750 Hz and 8000 Hz in the tinnitus group within ROI, the comparisons between these auditory stimulations and ISR were not significant (Figures 5(a) and 5(b)). Conversely, HbO responses to 750 Hz and 8000 Hz decreased in the non-ROI (750 Hz, *t* = −3.38, *p* < 0.001; 8000 Hz, *t* = −2.14, *p* = 0.04; Figures 5(c) and 5(d)).

**Figure 6:**
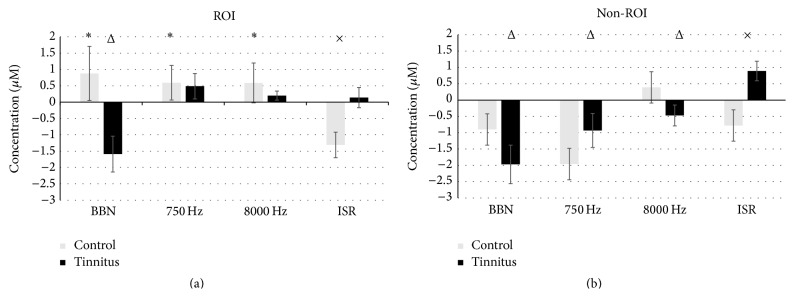
Bar graph of quantified oxy-hemoglobin (HbO) concentration with standard errors in region of interest (ROI; (a)) and nonregion of interest (non-ROI; (b)) under conditions of silence (interstimulus rest; ISR) and stimulation (750 Hz, 8000 Hz, and broadband noise; BBN) between tinnitus (dark bars) and control (gray bars). The unit of concentration is micromolar (*μ*M). Standard errors of the mean were calculated using the equation: *σ*_*x*_ = *S*/√*N* with *S* = sample standard deviation; *N* = the number of values. Note the maintenance of HbO concentration in ROI (a) and the significant increase in non-ROI (b) during ISR in the tinnitus group as compared to control (^×^ *p* < 0.05 relative to tinnitus versus controls). Under conditions of stimulation, only BBN suppressed previously maintained HbO levels in tinnitus ROI (a), while all forms of auditory stimulation suppressed increased HbO during ISR in non-ROI (^Δ^ *p* < 0.05 relative to ISR in tinnitus). Under conditions of stimulation or rest, only the ROI (a) showed a difference between ISR and all forms of auditory stimulation (^*∗*^ *p* < 0.05 relative to control ISR) and only during ISR significant differences were found between tinnitus and control; ^×^ *p* < 0.05. Error bars represent average responses from 4–18 seconds recorded from all conditions across all groups.

The comparison between ROI and non-ROI during acoustic stimulation (750 Hz, 8000 Hz, and BBN) revealed a significant difference in response to 750 Hz (*t* = 3.7; *p* < 0.001) in controls and only a trend in response to 8000 Hz (*t* = 0.5; *p* = 0.6) and BBN (*t* = 1.01; *p* = 0.32), revealing the as expected result of increased auditory cortex activity following acoustic stimulations as compared to non-ROI. Similar results were found between ROI and non-ROI in tinnitus participants (750 Hz, *t* = 2.7, *p* = 0.01; 8000 Hz, *t* = 0.52, *p* = 0.61; BBN, *t* = −0.17, *p* = 0.86; Table 1).  Figure 6 graphically summarizes the changes in HbO concentration in both groups under all tested conditions.

## 4. Discussion

### 4.1. Increased Activity in Tinnitus at Rest

Our data demonstrated maintenance of elevated hemodynamic activity during periods of silence in tinnitus participants supporting our central hypothesis that auditory cortical regions are spontaneously active in the absence of stimulation as compared to nontinnitus controls. Animal models of noise-induced tinnitus demonstrate increased spontaneous and tone-evoked neural firing rates and enhanced neural synchrony in auditory cortex [5] and brainstem [37, 38]. Increased neuronal firing rates and enhanced synchrony have both been touted as putative physiologic correlates of tinnitus [5, 38]. However, caution should be exercised in equating animal models of tinnitus with the human condition, in particular given the strong emotional and cognitive aspect in humans that cannot be equated or demonstrated in an animal model.

When combined with cortical tonotopic map reorganization [39–41] and alterations between central auditory neural excitation and inhibition [8], the reported neural changes highlight plasticity within the central circuit that may underlie tinnitus etiology and ultimate phantom sound perception. Our fNIRS results showing alterations in waveforms representative of changes in neural hemodynamic properties in human tinnitus may directly reflect the basic science neural correlates. To our knowledge, only one other study utilizing fNIRS technology [18] has demonstrated the aberrant effects of tinnitus in the human brain while other neuroimaging studies have attempted to identify the equivalent purported basic science neural correlates from animal models. Lanting et al. [8] reviewed several PET and fMRI studies that demonstrated enhanced steady state neural activity across multiple central auditory centers as a potential correlate of tinnitus.

Our data are consistent with the findings that primary auditory regions maintained hemodynamic activity under periods of silence while control subjects showed a deactivation in hemodynamic response. The maintenance of hemodynamic activity within primary auditory regions may reflect increases in spontaneous neural discharge rates found reliably in animal models suggesting that the human tinnitus brain may also exhibit this physiologic change in the absence of an actual acoustic stimulus. Indeed, the perception of tinnitus is considered to be the result of increased neural (cortical, subcortical) activity. This can have two opposite types of effects on the magnitude of an evoked or event-related potential (ERP); one may hypothesize that in the presence of elevated tonotopic activity at tinnitus frequency, the magnitude of an ERP elicited by the same frequency may be increased as it is added to the existing synchronized activity. Conversely, the result could also be a reduced response due to the availability of fewer neurons to generate the synchronized activity necessary to generate an EP or ERP.

One unexpected finding in the present study is the elevated hemodynamic activity in non-ROI in tinnitus. While we show that human tinnitus may also influence nonauditory cortical regions, other imaging modalities have identified enhanced brain activity in regions outside auditory cortex, including areas dedicated to attention and emotion in tinnitus [8, 21]. Utilizing a single-photon emission computer tomography (SPECT) scan, which uses gamma rays and has the ability to provide 3D information, Shulman et al. [42] demonstrated significant abnormalities in cerebral perfusion in multiple brain regions including auditory, frontal, and parietal cortices in human tinnitus. Others have also demonstrated changes in multiple brain regions in individuals with tinnitus using EEG, including primary and secondary auditory cortices, anterior cingulate cortex, dorsal lateral prefrontal cortex, insula, parahippocampus, and posterior cingulate cortex [7, 43]. Elevated waveforms during silence in non-ROI in our data agree with data from other optical imaging modalities suggesting that potential maladaptive changes in tinnitus extend beyond primary auditory cortices to nonauditory areas. Our non-ROI channels corresponded to regions in occipital cortex, which has been identified by optical imaging studies in tinnitus brains to show altered processing [25–27, 44, 45]. Another maladaptive change identified in human tinnitus utilizing optical imaging is hemispheric asymmetry [8, 46–48]. Using PET technology, Langguth et al. [48] found that 17/20 tinnitus participants displayed increased activity in left versus the right auditory cortex suggesting that interhemispheric differences may contribute to tinnitus etiology or perception. Our analysis of waveforms from both ROI and non-ROI did not identify significant interhemispheric asymmetry in either group but did demonstrate an overall left greater thann right trend across both groups. While this may be, in part, a reflection in variability in handedness amongst the participants admitted limited power of this pilot study could explain the lack of statistical significance between hemispheres. Together, these data suggest that fNIRS as an imaging modality is capable of accurately measuring and discerning subtle and dynamic changes in cortical hemodynamic activity under conditions of stimulation and silence in both normal and aberrant neural circuits.

### 4.2. Neurophysiological Responses to Sound

The present results using fNIRs technology demonstrate differences in auditory and adjacent nonauditory cortical hemodynamic responses in humans with subjective tinnitus. Similar to Schecklmann et al. [18], we demonstrate the capability of fNIRS to investigate brain changes that may underlie tinnitus etiology. Our data show clear neurophysiological responses within the auditory cortex following acoustic stimulation in tinnitus participants as compared to regions not primarily associated with auditory processing. Additionally, these results were also comparable with those of normal hearing controls and consistent with our predictions and previous reports utilizing alternative optical imaging modalities. Indeed, the data showed a canonical hemodynamic response in ROI with auditory stimulation and lack of response in non-ROI under the same conditions [13–17, 23].

Although increased tone-evoked firing rates to auditory stimuli are touted in animal models as potential neural correlates of tinnitus [5], our data did not demonstrate increased hemodynamic responses to auditory stimuli in tinnitus participants. This may be a relevant demonstration of the differences between tinnitus perceived by humans and animal models. While animal models are important in demonstrating neurophysiological phenomena in tinnitus, they have limitations in terms of describing the human condition. Moreover, these results agree with findings from Attias [49] using other optical imaging techniques on humans, suggesting that this abnormal activity is unlikely to underpin tinnitus perception [49–52]. Interestingly, BBN significantly decreased hemodynamic responses in both ROI and non-ROI within the tinnitus group. This stands in contrast to controls, where an expected elevated response in ROI and not in non-ROI was seen. This suppression of cortical activation potentially represents forward masking and based on the permanence of temporal effect, also residual inhibition, where an external sound is used to suppress “phantom” perception. Forward masking is thought to work through the interruption of abnormal synchronous activity among networks of neurons that generate tinnitus by the external sound stimulus [53]. Capitalizing on the well-recognized phenomenon of tinnitus modulation in certain participants, others have demonstrated with optical imaging techniques that tinnitus perception is associated with changes in activity within the auditory circuit [50, 54, 55]. Melcher et al. [56] utilized fMRI with and without sound from the scanner pump to demonstrate that hemodynamic responses to auditory stimuli in tinnitus were blunted when the pumps were on relative to when the pumps were off, a finding absent in controls. Mirz et al. [57] utilized PET imaging during habitual and blunted tinnitus sensation, reporting that suppressing tinnitus perception decreased cortical activity. The decreased activity seen in our tinnitus group in response to BBN may reflect similar phenomena. Future studies will be required to assess tinnitus perceptual changes during prolonged experimental paradigm to closer investigate residual inhibition. A longer block paradigm would also provide better insight into both initial and sustained hemodynamic responses to auditory stimuli as well as reducing confounding factors of the cortical measurements in various experimental conditions.

### 4.3. Limitations

A limitation of this study is the interpretability of optical imaging data from participants with heterogeneous brains. Spatial normalization is an image processing step used with other optical imaging modalities, such as fMRI or PET, to help standardize optical imaging data given the variability in human participant brain size and morphology. We did not utilize that approach as that would require the use of a second optical imaging modality. While many published fNIRS reports have not implemented this technique as a standard practice in data collection, future studies will be designed to incorporate multiple optical imaging modalities.

A potential limitation to the study is the statically significant difference in the age of the two groups. This difference may influence the differential path length (DPF) that depends upon the proportions of different scatterers and absorbers in tissue, including the presence of bone, myelin, and muscle [58]. Although these proportions can be heterogeneous within any study group, DPF has been found to be approximately constant with the use of a source detector distance over 2.5 cm [59–61]. With aging, there are changes in tissue properties that may alter the DPF. Duncan et al. [58] assessed differences in DPF at a fixed frequency and a source-detector separation for 283 subjects from 1 day to 50 years of age, finding that there is a slowly varying age dependence of DPF across all the study subjects. Modeling studies have shown that an increase in scattering or lowering in absorption can lead to an increase in DPF [62]; the higher DPF values seen with increases in age are expected given the increases in myelin that occurs with aging. When analyzing DPF values of only adult subjects, Duncan et al. [63] found no correlation between DPF and age within this subgroup existing. Given that all of our study subjects were adults, we feel that ultimately we had a consistent DPF in our study and essentially void of any bias form differences in age across the subjects.

Another limitation of the current study is the relatively small number of subjects in both groups. Despite this limitation, the current responses are quite compelling and provide a solid foundation for future, potential multicentered trials to test more complex variables in this population. We also acknowledge that the reduced numbers also limit our ability to correlate tinnitus severity (per the testing index) and age differences with hemodynamic response. This will certainly be important going forward as fNIRS could then have potential clinical diagnostic and potentially prognostic application. Further limitation of this study includes the use of a fixed DPF value for both wavelengths. As demonstrated by Strangman et al. [64] and Boas et al. [65], the use of the same DPF for 690 and 830 nm may lead to cross-talk in the estimates of HbO and HbR concentrations. Future studies should consider cautiously the appropriate value for each wavelength in order to reduce systematic errors.

## 5. Conclusions

The present study uses fNIRS technology to investigate and report hemodynamic response changes that occur in auditory and select nonauditory cortices in the human brain in those suffering from subjective tinnitus. Our data support the hypothesis that auditory cortices maintain and even increase hemodynamic activity during periods of silence, supporting, and even potentially representing putative neural correlates of tinnitus from animal models implicating increased spontaneous neural firing rates as a potential underlying etiology. While the measured changes in hemodynamic activity using fNIRS may not be a direct reflection of physiologic correlates, our results support future application of noninvasive fNIRS technology to further investigate translational correlates of central auditory aberrancy.

## Figures and Tables

**Table 1:** Mean oxy-hemoglobin (HbO) concentration and standard error (parentheses) from region (ROI) and nonregion of interest (non-ROI) during silence (interstimulus rest; ISR) and stimulation (750 Hz, 8000 Hz, broadband noise; BBN) between tinnitus and control. The unit of concentration is micromolar (*μ*M). Independent and pairwise *t*-test analyses were conducted as shown to assess hemodynamic response differences during the various experimental conditions within and between groups for all conditions. Relative values to zero reflect a hemodynamic response deactivation (negative) versus activation (positive) in hemodynamic response (statistically significant as compared to ISR in controls^*∗*^; as compared to ISR in tinnitus^Δ^; as compared to BBN in tinnitus^Ω^; and between tinnitus and controls^×^; *p* < 0.05).

HbO	BBN	750 Hz	8000 Hz	ISR
ROI				
Control	0.88 (0.83)^*∗*^	0.595 (0.53)^*∗*^	0.59 (0.61)^*∗*^	−1.31 (0.39)
Tinnitus	−1.59 (0.55)^Δ^	0.49 (0.39)^Ω^	0.20 (0.14)^Ω^	0.14 (0.31)^×^

Non-ROI				
Control	−0.90 (1.27)	−1.96 (0.63)	0.39 (0.75)	−0.78 (0.26)
Tinnitus	−1.97 (0.59)^Δ^	−0.93 (0.52)^Δ^	−0.47 (0.32)^Δ^	0.89 (0.30)^×^
